# Characteristics of Resistive PM Sensors for Onboard Diagnostics of Diesel Particulate Filter Failure

**DOI:** 10.3390/s22103767

**Published:** 2022-05-16

**Authors:** Kwang Chul Oh, Kyoung Bok Lee, Byeong Gyu Jeong

**Affiliations:** Korea Automotive Technology Institute, Cheonan-si 31214, Korea; kblee@katech.re.kr (K.B.L.); jeongbg@katech.re.kr (B.G.J.)

**Keywords:** particulate matter, diesel particulate filter, onboard diagnostics, PM sensor, DPF monitoring, diesel engine, resistive sensor

## Abstract

In accordance with the recently reinforced exhaust regulations and onboard diagnostics regulations, it is essential to adopt diesel particulate filter systems in diesel vehicles; a sensor that directly measures particulate matter (PM) in exhaust gas is installed to precisely monitor diesel particulate filter (DPF) failure. Because the reduction of particulate matter in the diesel particulate filter system is greatly influenced by the physical wall structure of the substrate, the presence or absence of damage to the substrate wall (cracks or local melting, etc.) determines the reliability of normal DPF operation. Therefore, an onboard diagnostics sensor for particle matter is being developed with a focus on monitoring damage to the DPF wall. In this study, as a sensor for determining damage to the substrate wall, an accumulation-type sensor whose resistance changes as soot particles are deposited between two electrodes was fabricated. The sensor characteristics were investigated by changing the gap between the sensor electrodes, sensor cap shape, and electrode bias voltage to improve resistive soot sensor sensitivity and response. From the signal characteristics of various sensor configurations, a combination sensor with improved signal stability and response time is manufactured, and they were compared with the characteristics of commercially available sensors in the engine-simulated NEDC mode in terms of the degree of DPF crack. As a result of transient mode, PM monitoring cycle was improved by 1.2~1.5 times during the same vehicle driving time compared to the existing commercial sensor.

## 1. Introduction

In general, diesel engines offer higher output and fuel efficiency, as well as lower carbon dioxide emissions compared to gasoline engines. However, in a diesel engine with compression-ignition-type combustion characteristics, a local fuel-rich region and a high-temperature flame region coexist in the combustion chamber, and for this reason, particulate matter (PM) and nitrogen oxide (NOx) emissions of diesel engines are high [1,2,3]. To solve this problem, combustion within the engine has been improved or technologies, such as high-pressure fuel injection and exhaust gas recirculation (EGR) have been developed. However, the ever-tightening emission regulations cannot be met with engine technologies alone. For this reason, many automobile-related companies and research institutes have actively been researching after-treatment systems for reducing the PM and NOx emissions of vehicles [4,5].

The use of a diesel particulate filter for PM reduction in the after-treatment system has become essential in light-duty diesel vehicles after implementation of the Euro-4 emission regulation (2004), and DPFs are now used in all diesel vehicles (DPF: more than 90% PM reduction) [6]. In the case of PM, it is completely dependent on the DPF system. Moreover, depending on the presence or absence of damage to the DPF system, a vehicle’s PM emission level differs. If the DPF is defective, pollutant emissions may exceed the previous emission limit because existing engine exhaust gas reduction technologies (especially, EGR technology, low-temperature combustion, dual-loop EGR) focus on nitrogen oxide reduction [7]. Therefore, normal operation of the DPF system is vital for maintaining the vehicle emission level. Moreover, because real-time verification is required, regulations pertaining to onboard diagnostics (OBD) limits are being strengthened [8]. The PM OBD level specified in the previous regulation could be met through system modeling by using a temperature sensor and a differential pressure sensor, but the current level requires one to use a sensor for directly monitoring PM [8,9]. Therefore, many diesel vehicles designed to meet the Euro-6 OBD regulation are equipped with a PM sensor that monitors whether the DPF system is operating as intended [10].

Such OBD PM sensors can be divided into two groups based on the underlying measurement principle: the ones in which a signal is analyzed for a certain time period (accumulation type) and the ones in which measurement is performed in real time (real-time type). The optical [11,12,13] and radio frequency [14] method and induced charge method [15,16,17,18] are representative examples of real-time measurement methods. However, many difficulties are encountered in the practical application of these methods onboard a vehicle, such as device complexity and harsh installation environments in terms of vibration and temperature, and these technologies involve optical access, high impedance, low current, or high time-resolution, which drives increased complexity and presents new durability challenges [19]. In contrast, the accumulation-type sensors, such as (i) electrical resistance-type [20,21,22] and (ii) charge storage-type [23] sensors, have relatively simple structures and are suitable for use as onboard sensors in vehicles. Moreover, their reliability is high even in harsh environments, such as exhaust pipes, and their manufacturing costs are low. For these reasons, accumulation-type sensors have been commercialized and installed in many diesel vehicles (electric resistance sensor—Bosch, Delphi sensors) [21,23,24,25].

For diesel vehicles whose exhaust emission level is affected by the operation of the after-treatment system, the operation monitoring regulation of the after-treatment system is strengthened by not only strengthening the OBD emission regulation level but also increasing the minimum monitoring update rate (In-Use Performance Ratio, IUPR) [22,26,27]. Therefore, the new PM sensor was developed with an emphasis on enhanced response time to detect smaller amounts of PM and stronger poisoning resistance for increased durability [28]. The response time and sensitivity of the resistive PM sensor are closely related to the polarization voltage, so there have been many attempts to improve the sensor characteristics by selecting a certain polarization voltage during measurement depending on the shape of the particles [29] and studies on improving the response time by applying voltage on/off in the sensor operation process [25] or pre-polarization during regeneration [30]. However, in consideration of the characteristics of the resistive sensor in which the sensing time for DPF monitoring becomes longer as the OBD threshold decreases, further improvement of the sensor characteristics is required.

In this paper, as a sensor for determining damage to the DPF wall, an accumulation-type sensor whose resistance changes with the deposition of soot particles between its two electrodes is fabricated. The sensor characteristics are verified by changing the gap between the two sensor electrodes and sensor cap shape and changes in the electrode bias voltage. From the signal characteristics of various sensor configurations, a combination sensor with improved signal stability and response time is manufactured and was compared with the characteristics of commercially available sensors in the engine-simulated NEDC mode in terms of the degree of DPF crack.

## 2. Experimental Setup and Method

**Figure 1:**
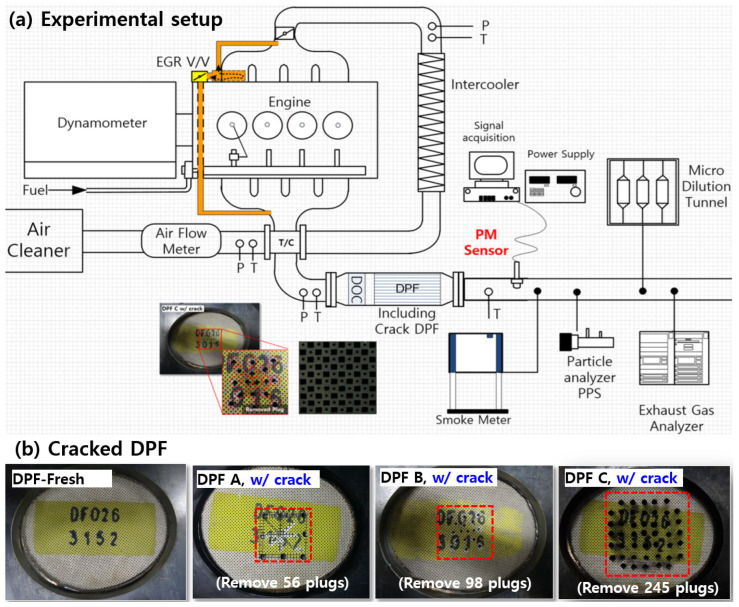
Experimental setup and cracked DPF, (**a**) experimental setup, (**b**) photos of Fresh and Cracked filters.

In this paper, a Euro 5 2.0-L diesel engine was used, as shown in Figure 1, to examine the characteristics of a fabricated resistance-change-type PM sensor. Sensor signal characteristics for various particle number concentrations were determined by removing the DPF and changing the EGR rate and engine operating point. The artificially damaged DPF thus obtained was used to determine sensor characteristics in the transient mode.

**Figure 2:**
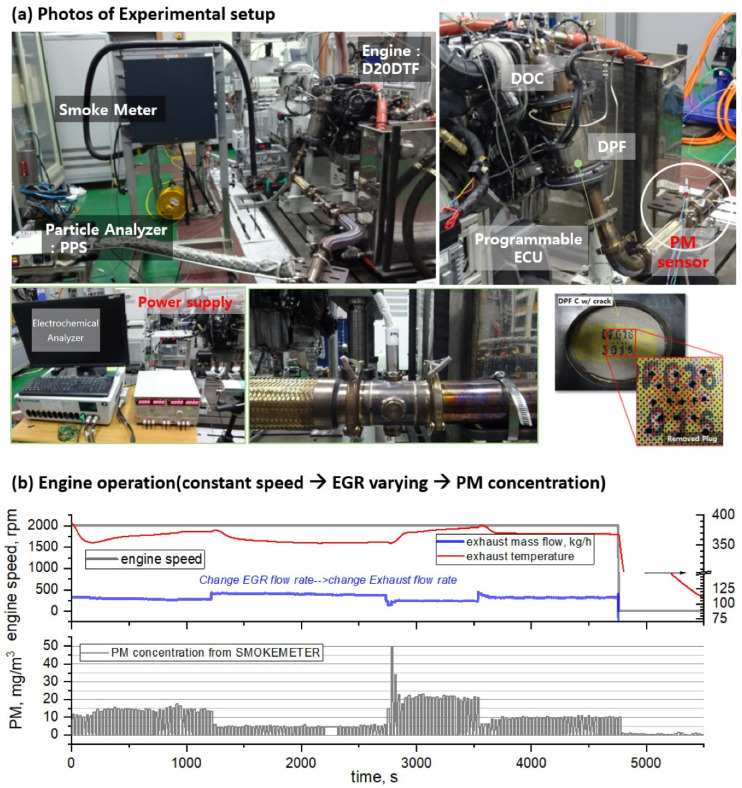
Photos of experimental setup and engine operation process for steady state test, (**a**) photos of Experimental setup, (**b**) procedure of engine operation.

Engine operation proceeds after preheating to stabilize PM emission, and PM concentration was controlled by changing EGR using a programmable engine control unit at the engine operating point, as shown in Figure 2. The exhaust flow rate of the engine was calculated by measuring the intake air flow rate and fuel consumption. In addition, a micro dilution tunnel (MDLT-1303, Horiba, Kyoto, Japan) and smoke meter (AVL 415S) were used for PM concentration, and a pegasor particle sensor (PPS-M, Pegasor, Tampere, Finland) was used to measure the number of particles. Table 1 shows engine information, after-treatment equipment, and fuel used in this study.

Table 2 shows the PM and particle number (PN) emissions of the artificially damaged filter, with the DPF plugging removed. Four DPFs were manufactured by removing the DPF plugging as shown in Figure 1b in consideration of the emission regulation level and OBD threshold in the NEDC mode. Fresh DPF (w/o crack) is within the PM emission regulation (4.5 mg/km), and DPF-A is higher than the emission regulation level and within the Euro-6 final OBD threshold (12 mg/km). Furthermore, DPF-B and C are filters that show emissions greater than the final threshold and are within and exceeding the primary threshold (25 mg/km), respectively.

### 2.1. Resistance-Change-Type PM Sensor

**Figure 3:**
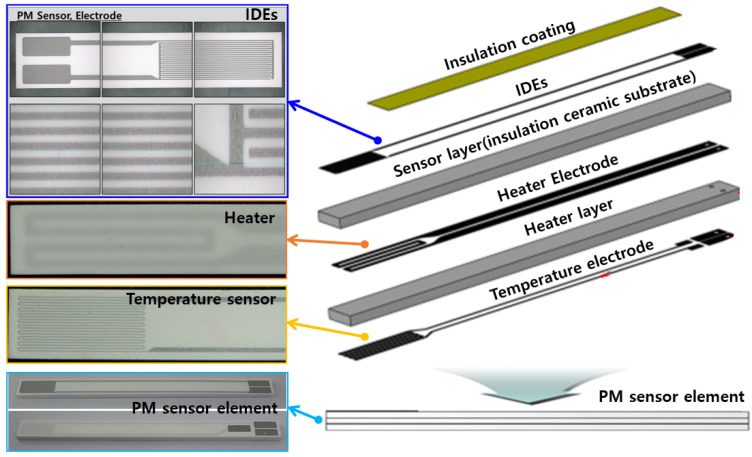
PM sensor element and components.

The resistance-change-type PM sensor used in this study consists of three layers, as shown in Figure 3. The top layer is a sensing electrode (interdigitated electrode), which is fabricated by alternating conductive metal oxides in a comb shape with a certain gap on a non-conductive alumina plate by means of screen printing.

The next layer is the heater electrode layer, which periodically raises the surface temperature of the sensing electrode to the oxidation temperature (600 °C or higher) to regenerate the PM accumulated on the sensing electrode. The last layer is fabricated by screen-printing a resistance temperature detector (RTD)-type temperature sensor as a temperature electrode.

**Figure 4:**
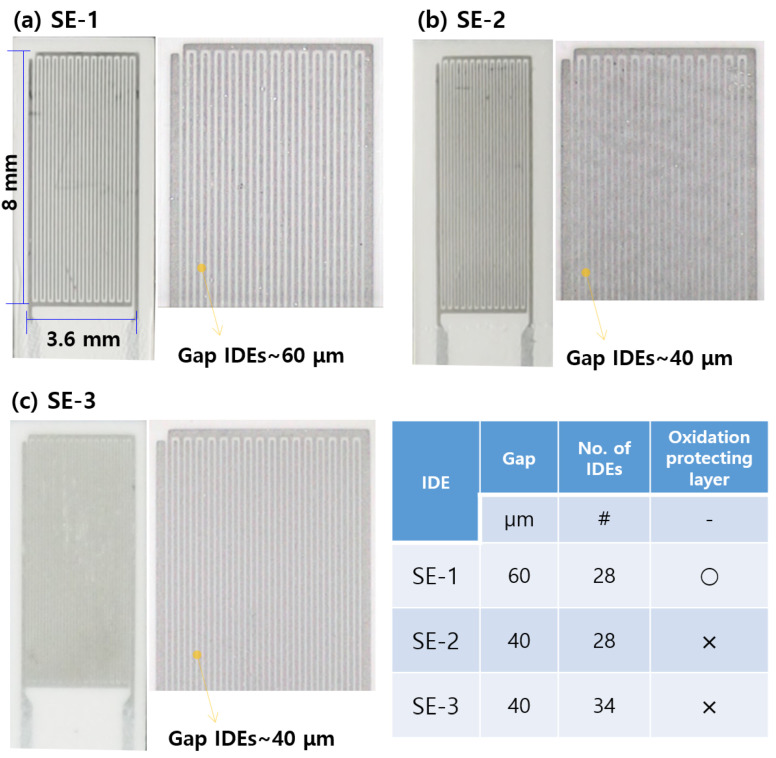
Shape of sensing IDEs, (**a**) SE-1, (**b**) SE-2, and (**c**) SE-3.

In this study, the signal characteristics’ three types of IDEs of different shapes (Figure 4) are compared. The sensing electrode measured 3.6 mm in width and 8 mm in length, and it was manufactured through screen printing on insulating ceramic (Al_2_O_3_) with a certain gap between the electrodes, drying, and simultaneous sintering.

**SE-1:** This electrode consists of 28 IDEs with an inter-IDE spacing of 60 μm, as well as an anti-oxidation layer.

**SE-2:** This electrode consists of 28 IDEs with an inter-IDE spacing of 40 μm, but there is no anti-oxidation layer

**SE-3:** This electrode consists of 34 IDEs with an inter-IDE spacing of 40 μm, and there is no anti-oxidation layer.

### 2.2. PM Sensor Packaging

**Figure 5:**
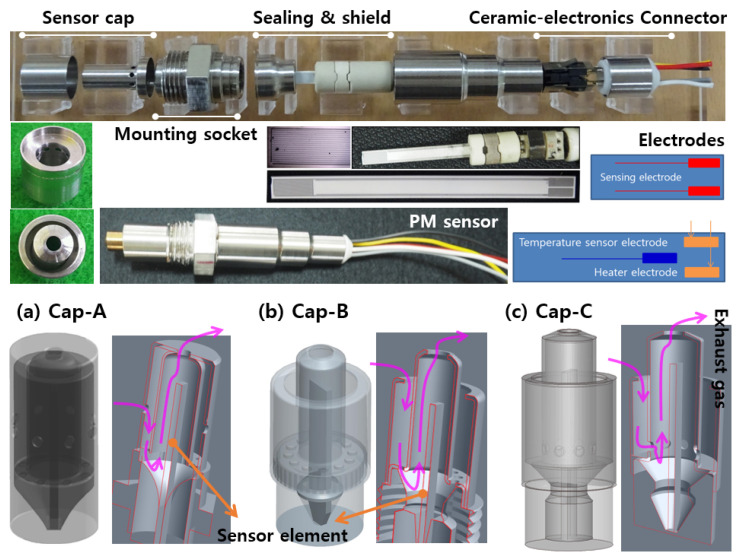
PM sensor packaging and sensor caps, (**a**) Cap-A: dual cap, (**b**) Cap-B: similar to Bosch cap, and (**c**) Cap-C.

The PM sensor element manufactured by following the stacking process was packaged as shown in Figure 5 and installed in an engine exhaust pipe. It consisted of a sensor cap part for separating the sensor element from exhaust gas and transferring a small portion of the exhaust gas to the sensing element, a part for coupling with the exhaust pipe, an exhaust gas sealing part, and a connector part for connecting the electrodes of the ceramic sensor to the controller.

Figure 5 shows the three types of sensor cap structures used in this study. Each sensor cap is divided into an inner cap and an outer cap, and the caps are designed such that some portion of the exhaust gas is introduced into the sensor without disturbing particle flow. In the structure of Cap-A, a portion of the exhaust gas flows in through the outer cap hole and is introduced to the sensor through the hole located under the inner cap. The internal inflow rate (Q_s_) of Cap-A is approximately 0.06% of the total exhaust flow rate (Q_exh_), which is close to half that of Cap-B’s 0.11%. In the case of Cap-C, by changing the flow path, the inflow volume is increased (Q_s_/Q_exh_~0.08) and PM accumulation inside the cap is prevented.

### 2.3. Engine Operation Condition and Mode

To observe the characteristics of the PM sensor as a function of the PM concentration and sensor flow rate, a test was performed in which the EGR flow rate at the NEDC representative operating point of the engine was varied. As shown in Figure 1 and Figure 2, a pegasor particle sensor (PPS-M, Pegasor), a smoke meter (AVL 415S), and a micro dilution tunnel (MDLT-1303, Horiba) were installed downstream of the DPF to check PM and PN emission characteristics. Moreover, the developed PM sensor was installed in the same position, and the sensor signal was acquired using an electrochemical analyzer (AUT128N, Autolab) that can measure micro-currents while maintaining the potential difference at the sensing electrode.

**Figure 6:**
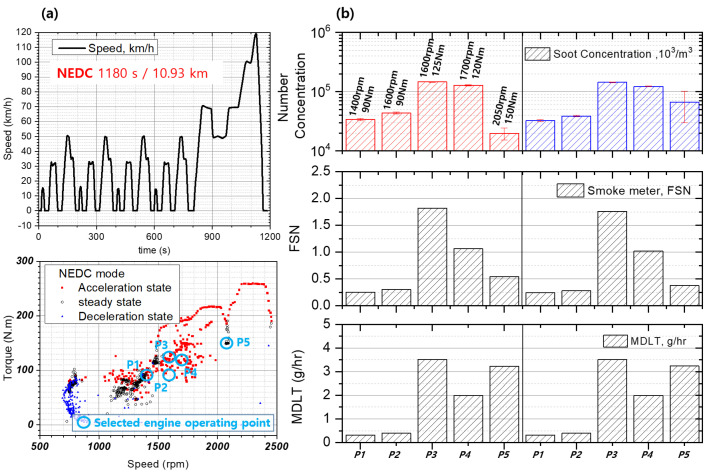
NEDC mode and PM emission at representative operating points in the NEDC mode, (**a**) NEDC mode and transient engine operation, (**b**) PM emission on selected engine operating points.

In order to examine the signal characteristics according to the change of sensor components, steady state engine operating points were selected in the NEDC mode range. Based on the selected operating point, the PM concentration was independently changed by changing the EGR while maintaining the similar range of exhaust flow rate and exhaust temperature. Figure 6 shows the NEDC mode used in this study and the PM emissions at the representative engine operating points in the NEDC.

## 3. Results and Discussion

The PM sensor signal changes depending on the shape of the sensing electrode and shape of the sensor cap, magnitude of bias voltage, and regeneration temperature. In this study, the signal characteristics were examined considering the three types of electrodes, three types of sensor caps, and bias voltage. Based on the results, a combination with good sensor sensitivity (response characteristics) was constructed, and the possibility of detecting filter cracks in the NEDC mode, a transient mode, was discussed.

### 3.1. Sensor Signal and Response Time

#### 3.1.1. PM Bridge Formation and Sensor Signal

**Figure 7:**
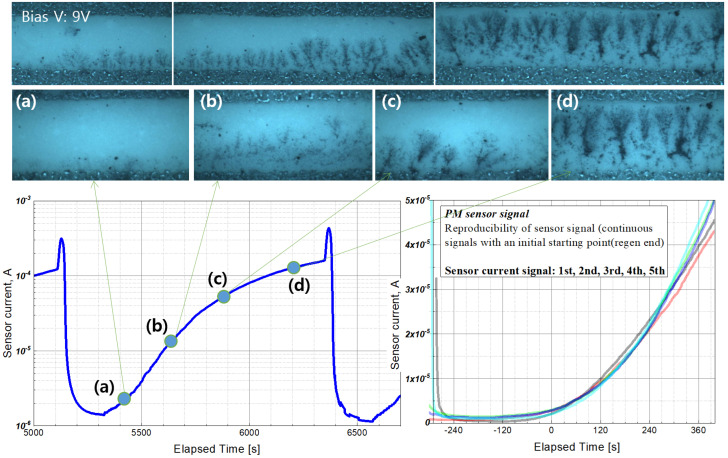
PM bridge formation and conventional sensor current signal at a bias voltage of 9 V and PM concentration of 20 mg/m^3^. (**a**–**d**) are positions of signal.

The typical signal characteristic of PM sensors is shown in Figure 7. The picture of Figure 7 was obtained using a microscope. A sensor using a detachable sensor cap was installed in the engine, and when the sensor signal reached a certain level, EGR was turned off, and the sensor surface was photographed using a microscope by detaching it from the engine exhaust. When the sensor is exposed to a certain PM concentration, the current through the sensing electrodes increases as PM is deposited between the electrodes. Electrophoresis of particles occurs when a voltage is applied between the two sensing electrodes and charged particles near the electrodes form a bridge between the electrodes [27,29]. The resistance of the sensing electrode decreases because of the bridge, and the current signal increases. Figure 7 shows the relationship of bridge formation between electrodes with the sensor signal.

If the electrical conductivity of the ceramic material (initial electrode resistance O (100) MΩ) is not considered at the beginning of sensor operation, a current signal is not obtained until a bridge is formed between the electrodes. As can be seen from the (a) picture of Figure 7, a bridge core is formed at a point near the electrode, and branches are spread in the direction of the opposite electrode with the core as the center. After that, it can be seen that a spot with high concentration is formed near the electrode and the branch grows. Thereafter, the sensor current gradually increases owing to the formation of a PM bridge between the electrodes, and the electrode is regenerated after the current threshold is reached or after the set sensing time has passed.

**Figure 8:**
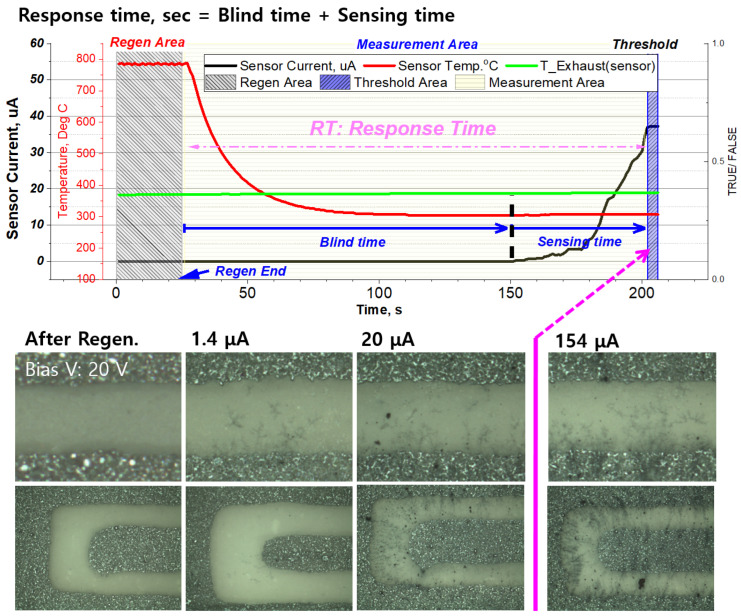
PM sensor response time definition, and bridge shapes to threshold at the bias voltage of 20 V and PM concentration of 10 mg/m^3^.

After the accumulated PM is regenerated by the heater, the sensor signal is not measured during the blind time, and when the PM concentration to which the sensor is exposed decreases, the blind-time increases [25,27,28,31]. As described above, when PM was deposited on the sensor and the threshold of the current signal was reached, power was applied to the heater to regenerate the sensor (about 800 °C.), and the signal characteristics of the sensor were examined for various PM concentrations. At this time, the retention time between regeneration and reaching the threshold was defined as the sensor response time (s) (Figure 8).

#### 3.1.2. Sensor Signal According to PM Concentration

**Figure 9:**
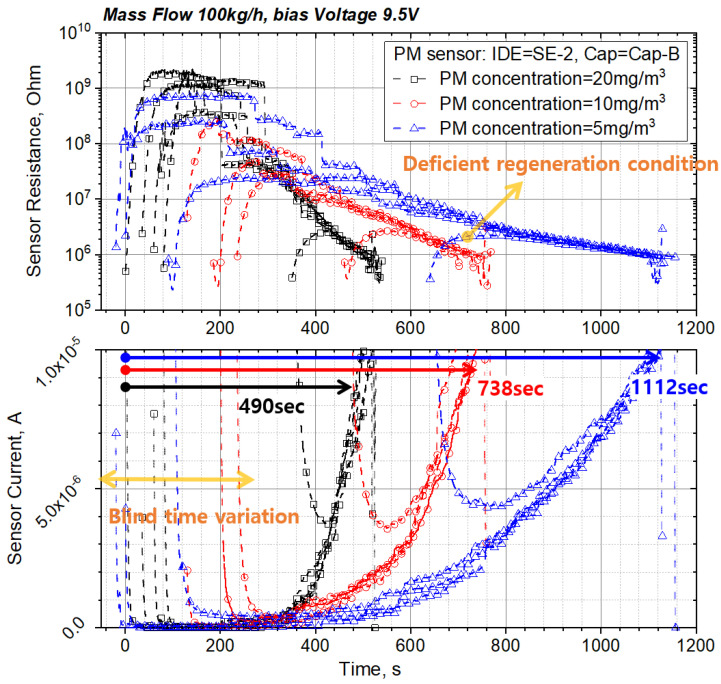
PM sensor signal and response time according to PM concentration at bias voltage of 9.5 V, sensor Cap-B, and IDE shape SE-2.

Figure 9 shows the sensor signal according to the PM concentration when the electrode shape is SE-2 and the sensor cap is Cap-B. In the case of 5 mg/m^3^ with the lowest PM concentration, the signal characteristics of the sensor change depending on the degree of regeneration. When the sensing electrode is not completely regenerated because the regeneration time is short, the bridge from the previous sensing operation remains, and the blind time decreases. The cause of this phenomenon can be inferred by looking at the PM bridge formation process in Figure 7 and Figure 8. If regeneration is insufficient or appropriate, PM seeds remain around the electrode or inside the electrode, so it is inferred that it helps to generate a core near the electrode during measurement and accelerates branch growth. This reduction of blind time leads to a change in the response time, which causes errors in crack detection. Therefore, the regeneration temperature and time required for complete electrode regeneration must be ensured, and it is important to set the initial current value at the bias voltage after regeneration. Based on the fully regenerated signal, the sensor response times (time required for the sensor current value to reach 10 μA) for the PM concentrations of 5, 10, and 20 mg/m^3^ decrease exponentially to 1112, 738, and 490 s, respectively.

### 3.2. Effect of Sensing Electrode Shape

**Figure 10:**
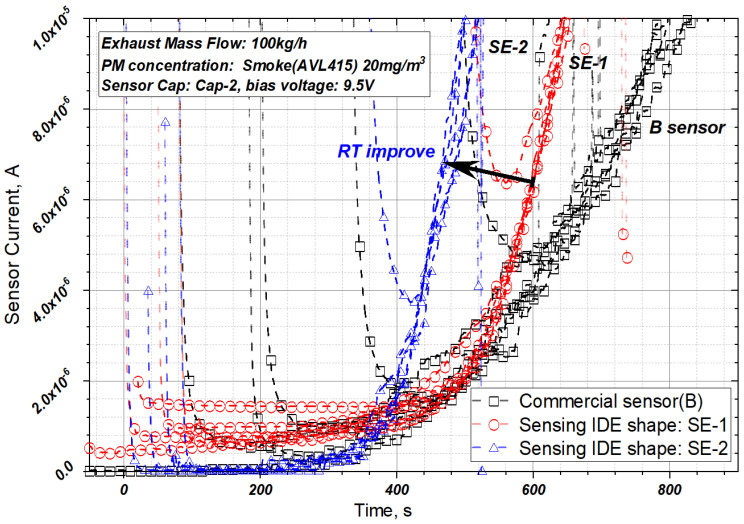
PM sensor signal according to shape of sensing IDE (SE-1/2) at the bias voltage of 9.5 V sensor Cap-B.

In this study, the effects of the shapes of the three sensing electrodes on the sensor signal were investigated. Figure 10 shows the signal obtained using sensor electrodes of different shapes when the bias voltage is 9.5 V. SE-1 and SE-2 have higher sensitivities compared to the commercial sensor, although there is a tendency depending on the degree of regeneration. SE-1, which has an anti-oxidation layer, requires more time to regenerate, and the resistance between its electrodes is low for the same regeneration time because of the larger IDE gap than that of SE-2, as shown in Figure 10. SE-2 has superior sensitivity and signal reproducibility compared to SE-1 owing to the reduced inter-electrode gap.

**Figure 11:**
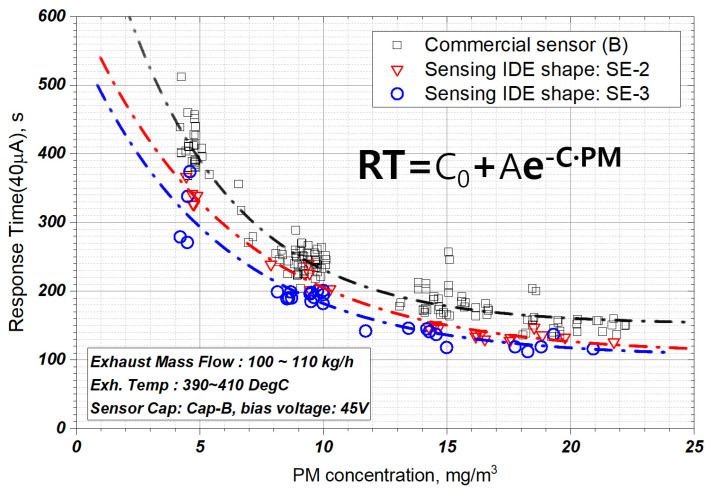
PM sensor response time according to sensing IDE shape at the bias voltage of 45 V and sensor Cap-B.

Figure 11 shows the signal characteristics according to the sensor electrode shape at the bias voltage of 45 V. Considering that the SE-2 electrode shape, illustrated in Figure 10, has a response time of up to 500 s at 10 μA, the response time of the signal improves greatly as the bias voltage increases (9.5 V–>45 V). In the case of SE-3, in which the inter-electrode spacing is maintained and the number of electrodes is increased, the response characteristics improve by approximately 10% in the PM concentration range of 5–25 mg/m^3^ compared to those of SE-2. This can be ascribed to the increase in the number of electrodes. Moreover, and the amount of PM required for bridge formation increases, meaning accuracy can be increased by performing measurements using larger sensor current values.

### 3.3. Effect of Bias Voltage

**Figure 12:**
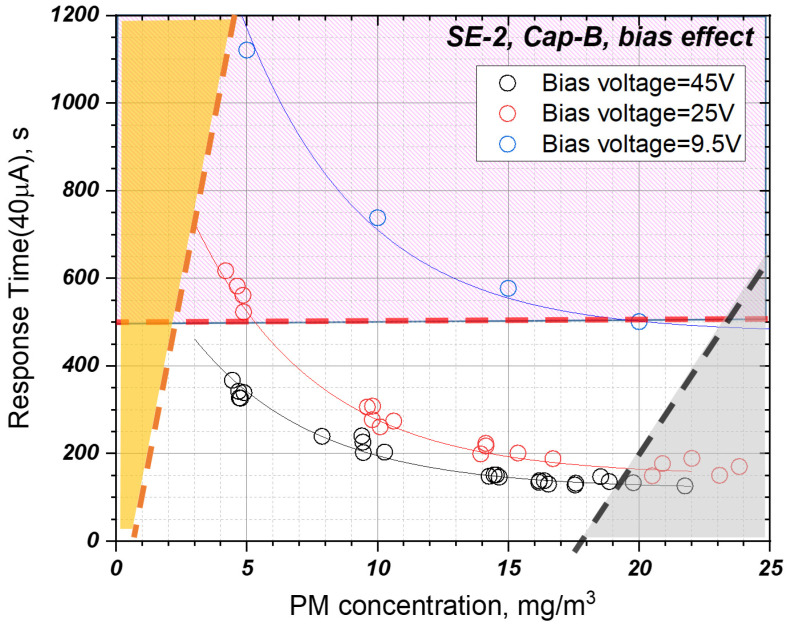
Sensor response time according to changes in bias voltage and PM concentration for SE-2 and sensor Cap-B.

Figure 12 shows the response characteristics of the sensor according to the PM concentration and the bias voltage applied to the sensor electrode. The sensor response time decreases as the PM concentration in the exhaust gas increases, and the response time decreases as the bias voltage applied to the sensor electrodes increases. In particular, the increase of the bias voltage greatly reduces the response time at the low PM concentration compared to the high PM concentration. This occurs because, as the bias voltage supplied to the sensor increases, the electrophoretic effect generated at both electrodes of the sensor increases, and thus, a bridge between the electrodes is formed quickly in the initial stages [29]. Therefore, it can be seen that it is an important means for shortening the sensing cycle when the DPF OBD threshold is decreased, and the monitoring update rate is increased in future vehicle driving conditions.

### 3.4. Effect of Sensor Cap

**Figure 13:**
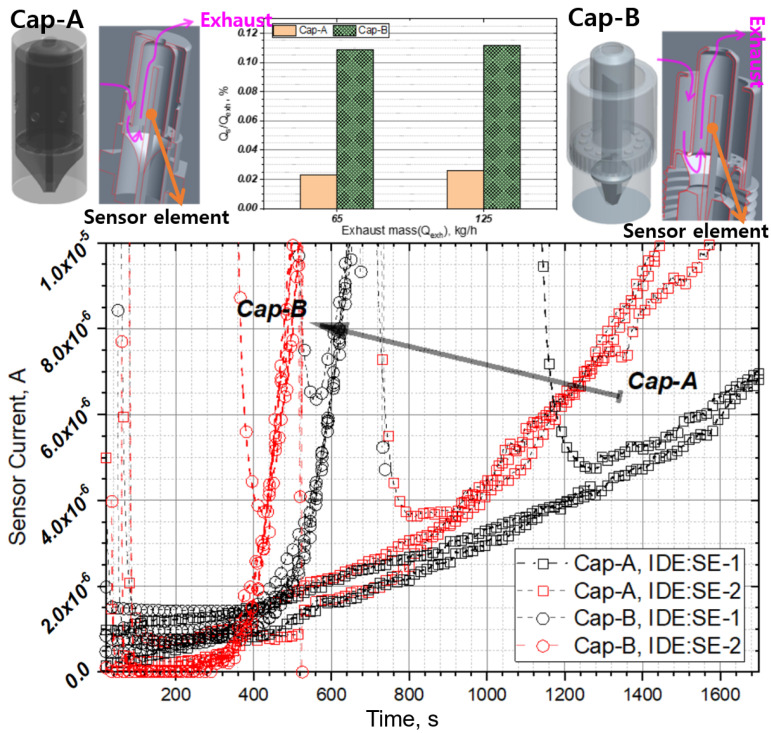
Sensor signals corresponding to different sensor cap types and IDE shapes at PM concentration of 20 mg/m^3^ and bias voltage of 9.5 V.

Figure 13 shows the signal characteristics for various types of sensor caps at the PM concentration of 20 mg/m^3^ and bias voltage of 9.5 V. When the sensor cap is changed to Cap-B, the signal response changes substantially because the exhaust gas inflow of Cap-A is significantly lower than that of Cap-B (Cap-A is ~1/5 of that of Cap-B) owing to the small outer cap hole of sensor Cap-A.

**Figure 14:**
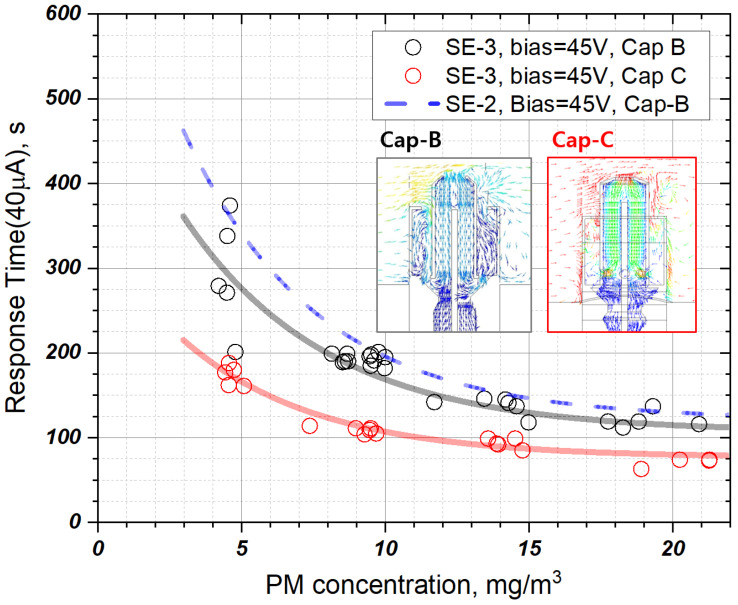
Comparison of sensor signals for various cap shapes and sensor elements at the bias voltage of 45 V.

The inflow rate of Cap-C is smaller than that of Cap-B. However, in Cap-C, the inflow hole inside the sensor is close to the sensor electrode. Because the flow pattern in the direction of the sensor element surface is used, contamination inside the sensor can be minimized compared to that in Cap-B, and if the relative position of the sensing electrode and the hole is maintained to some extent, initial signal stability and sensor response time can be improved. Figure 14 shows the comparison results of sensor response characteristics according to PM concentration obtained by applying Cap-B and Cap-C to the SE-3 electrode shape. When Cap-C is applied, the response speed is improved by 40% at low PM concentrations and 30% at high PM concentrations compared to those when Cap-B is applied. By contrast, it is difficult to obtain signal sensitivity in high-concentration regions.

### 3.5. DPF Crack Monitoring in Transient Mode

The performance of the developed sensor was compared to that of the commercially available sensor based on DPF cracking in the transient mode. In terms of configuration, the developed sensor consisted of an SE-3 sensing element, 34 electrodes, and inter-electrode spacing of 40 μm, and the sensor cap was Cap-C, which has the fastest response time when considering the OBD regulation threshold and monitoring rate.

**Figure 15:**
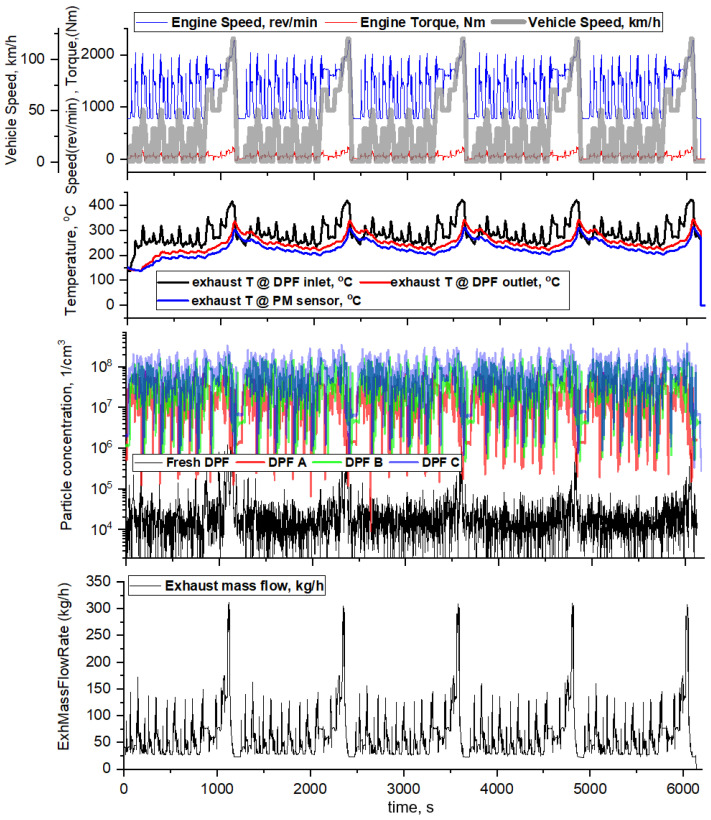
The engine operating conditions, exhaust temperature, PN emission, and exhaust flow rate in engine-simulated NEDC mode.

The transient test was performed by repeating the NEDC engine-simulated mode five times, and the DPF was changed in the order of fresh DPF/DPF A/DPF B/DPF C based on the degree of crack. Figure 15 shows the engine operating conditions, exhaust temperature, and PN emission measured at the DPF outlet when the engine simulation NEDC mode is continuously performed five times. The exhaust temperature at the PM sensor position changes in the range of 200–300 °C, and as the transient mode progresses, the exhaust flow fluctuates from 20 kg/h in IDLE to 310 kg/h in the high-speed region. In addition, the emission of particulate matter varies depending on the state of the DPF. Fresh DPF without cracks has a concentration of 10^4^~10^5^ #/cm^3^, and it can be seen that PN emission increases according to the degree of cracks in DPFs A, B, and C, respectively.

**Figure 16:**
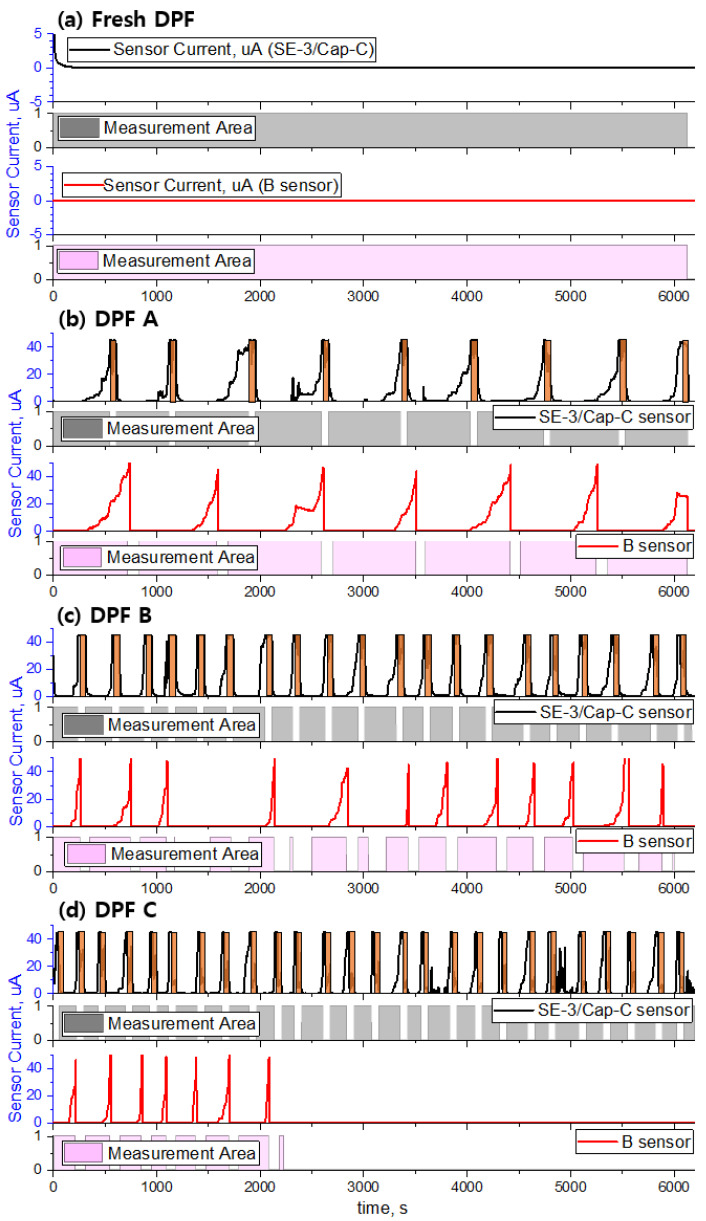
DPF crack monitoring with developed sensor and commercial sensor in NEDC mode, (**a**) Fresh DPF, (**b**) DPF A-cracked filter, (**c**) DPF B-cracked filter, (**d**) DPF C-cracked filter.

Figure 16 shows the measured sensor signal according to the degree of DPF cracking. During repeated executions of the NEDC mode, as the degree of DPF cracking increases, the response time of the sensor decreases, and the number of failure diagnoses increases. However, in the case of fresh DPF, the average concentration of discharged PM was less than 0.1 mg/m^3^, which is extremely low, and for this reason, no change in the sensor signal was observed. This implies that the PM concentration in the exhaust was extremely low, and consequently, bridge formation between the sensor electrodes did not occur.

In the case of DPF A, which is larger than the Euro-6 PM emission regulation value (4.5 mg/km) and smaller than the OBD threshold (12 mg/km), the developed sensor was able to complete approximately 1.8 sensing cycles per mode, which is 1.3 times higher than the 1.4 sensing cycles per mode of the commercial sensor. In addition, in the cases of DPFs B and C with cracks larger than the OBD regulation level, the sensing cycles per mode were 4 and 5.2, respectively. This result implies that it is possible to monitor the damage of the DPF frequently and reliably because the sensor can perform many sensing cycles at the same time through the optimization of the electrode, bias voltage, and sensor cap. As summarized in Table 3, the number of sensing cycles and the response time of the developed sensor change depending on the degree of DPF cracking.

## 4. Conclusions

Herein, the authors explained the signal characteristics of the resistive PM sensor and the DPF damage diagnosis principle. Moreover, the authors investigated sensor characteristics according to the sensor element, sensor cap, bias voltage change, and the emitted PM concentration. In addition, by using DPFs with various degrees of failure in the transient mode, the authors confirmed that the developed sensor can detect DPF failure well, relative to a commercially available sensor.

(1) As the PM emissions decreased at the engine steady operating point, the response time of the PM sensor increased exponentially. This implied that bridge formation between the sensor electrodes was delayed, and the blind time increased.
(2) The sensor response characteristics to the sensor pattern shape, electrode spacing, number of electrodes, and bias voltage between the electrodes were investigated. Among them, changes in the bias voltage supplied to the sensor element led to dramatic changes in the sensor response characteristics. This occurred because, as the bias voltage supplied to the sensor increased, the electrophoretic effect generated at both sensor electrodes intensified, leading to rapid bridge formation between the electrodes in the initial stage.
(3) In sensor Cap-C, when the distance between the sensor electrode and the inner hole was short, the signal was unstable owing to the interference caused by the attachment and detachment of particles during initial bridge formation as a result of the interference between the electrode and collision flow. However, the signal was stable and response time improved when the distance between the sensor electrode and the inner hole was maintained.
(4) The signal characteristics and reproducibility of the PM sensor, which shortened the IDE gap (40 μm), increased the bias voltage (45 V), and changed the flow pattern to the sensor element with the sensor cap (Cap-C) according to the PM concentration in NEDC mode, were investigated by using cracked DPFs. In terms of sensor performance, the response time of the developed sensor was faster than that of the commercially available sensor. Moreover, the number of sensing cycles of the developed sensor was 1.2–1.5 times higher, on average.

## Figures and Tables

**Table 1:** Engine and after-treatment specification.

Engine Name	D20DTF	EGR and Fuel	
Displacement (cc)	1998	EGR	Water cooled HP EGR
Cylinder No.	4	Charge cooling	Intercooler
Combustion cycle	4 stroke	Fuel system	CRDI
Method of aspiration	TCI(VGT)	Fuel	ULSD
Max. power (PS/rpm)	181/4000	**After-treatment system**	
Max. speed (rpm)	4600	DOC	0.9 L
Emission certification	Euro-5	DPF	Oval, 2.5 L

**Table 2:** Emission level of cracked DPF filter in NEDC mode.

Case-DPF	PN, #/km	PM, mg/km
**Fresh-DPF**	7.9 × 10^10^	0.02
**DPF A w/crack**	3.5 × 10^13^	7.00
**DPF B w/crack**	8.5 × 10^13^	17.11
**DPF C w/crack**	1.4 × 10^14^	28.30

**Table 3:** Number of sensing cycles and range of RT according to DPF cracking (Sensor: SE-3/Cap-C/bias = 45 V).

Case-DPF	PN #/km	PM mg/km	Number of Sensing Cycle #/NEDC	RT Range s
**Fresh-DPF**	7.9 × 10^10^	0.02	-	-
**DPF A**	3.5 × 10^13^	7.00	1.8	500–900
**DPF B**	8.5 × 10^13^	17.11	4.0	400–200
**DPF C**	1.4 × 10^14^	28.30	5.2	200–100

## Data Availability

Not applicable.

## Nomenclature

Q	flow: g/s
T	temperature, °C
P	pressure, mbar
R	resistance, Ω
RT	response time, s
DPF	Diesel Particulate Filter
NEDC	New European Driving Cycle
LTC	Low-temperature Combustion
PN	Particle Number
PM	Particulate Matter mass
EGR	Exhaust Gas Recirculation
OBD	Onboard Diagnostics
IDEs	Interdigitated Electrodes
Subscripts	
s	sensor
exh	exhaust
